# Birth Outcomes and Natural Gas Development: Methodological Limitations

**DOI:** 10.1289/ehp.1408647

**Published:** 2014-09-01

**Authors:** Kristen Fedak, Sherilyn Gross, Megan Jacobsen, Brooke Tvermoes

**Affiliations:** Cardno ChemRisk, Boulder, Colorado, USA

We read with interest the article “Birth Outcomes and Maternal Residential Proximity to Natural Gas Development in Rural Colorado” by McKenzie et al. (2014). We agree with the authors that it is important to determine whether any adverse health effects may be associated with active natural gas wells, especially for susceptible subpopulations.

On the basis of the prevalence of neural tube and congenital heart defects reported in infants born to mothers that lived within a 10-mile radius of natural gas wells, McKenzie et al. (2014) reported an association between natural gas development and these specific birth defects. They suggested that “potential teratogens”—particularly benzene and polycyclic aromatic hydrocarbons—emitted from the wells, related infrastructures, or drilling processes could be a causal factor related to the health effects. They generally weaved a cautionary tale regarding natural gas development and negative reproductive/developmental consequences. Although we applaud the authors’ efforts to investigate potential human health concerns related to oil and gas development, we would like to highlight key weaknesses within the study design that were underemphasized in their article. Specifically, we believe that the chosen exposure metric—inverse distance weighted gas well counts in a 10-mile radius of maternal residence during the child’s birth year—is a poor surrogate for an actual (i.e., chemical) exposure that might be causally linked to the outcomes of interest, which severely limits the ability to interpret results. In addition, the exposure metric raises issues regarding the biologic plausibility of benzene as the underlying causal agent for the observed effects.

McKenzie et al. (2014) used the gas well density and the distance of gas wells to maternal residence as a proxy for maternal chemical exposure, including in their count any well listed as “existing” within the Colorado Oil and Gas Information System (COGIS) registry during the entire birth year of the infant. However, the neural plate and heart are known to develop during the first trimester, and it is recognized that this is the critical period of sensitivity for induction of defects due to toxicological insult (Rogers and Kavlock 2008). Thus, it would be more appropriate to limit the maternal exposure metric to a window representative of only the first trimester of pregnancy. Additionally, the first trimester of a pregnancy may fall in a different calendar year than the child’s birth. Therefore, the authors may have inaccurately characterized maternal exposures for most subjects and severely misrepresented exposures for some. Although McKenzie et al. briefly noted in their “Discussion” that there was insufficient data to determine well counts tied to trimesters as opposed to birth year, we feel that this limitation is understated considering the potential impact.

We have previously determined that benzene is a highly volatile compound with a short atmospheric residence time and is unlikely to travel long distances from the emission source [Voluntary Children’s Chemical Evaluation Program (VCCEP) 2006]; the most relevant benzene exposures occur from nearby sources [Agency for Toxic Substances and Disease Registry (ATSDR) 2007; VCCEP 2006]. Interestingly, when McKenzie et al.’s analysis was restricted to wells within a closer proximity (e.g., 1- and 5-mile radii), results were not significant, leading one to question whether the reported results are truly indicative of a causal relationship or simply an artifact of arbitrarily selected parameters. Further, there are many inactive wells on the COGIS registry, which have different benzene emissions than active wells, a distinction that was not captured by the exposure metric. Moreover, McKenzie et al. implied that a causal link between benzene and congenital heart defects has been established and therefore their exposure proxy is justified—although the cited references do not actually provide such evidence, and current consensus documents do not recognize such an association [ATSDR 2007; International Agency for Research on Cancer (IARC) 2012; Lupo et al. 2010; VCCEP 2006; Wennborg et al. 2005).

McKenzie et al. (2014) acknowledged in their article that there is a “lack of temporal and spatial specificity” in their exposure metric, but they appear to primarily relate this to uncertainties such as potential maternal mobility and relative well activity levels. These are minor concerns compared with the larger issue of whether the chemical of interest and the parameters chosen were meaningful, appropriate, and well categorized. As scientists, we have an obligation to appropriately and effectively communicate to the public not just positive and negative findings but also some sense of the magnitude of risk in order to ensure that we do not create or perpetuate an unnecessary level of alarm. Based on the inherent limitations of this study, including that no true exposure to any chemical was actually measured or modeled and that the proxy exposure metric was weak, the suggested association between specific birth defects and natural gas exploration and production reported by McKenzie et al. (2014) should be viewed cautiously and critically.
